# Chemopreventive Effects of Silibinin on Colitis-Associated Tumorigenesis by Inhibiting IL-6/STAT3 Signaling Pathway

**DOI:** 10.1155/2018/1562010

**Published:** 2018-10-25

**Authors:** Rongjuan Zheng, Jiaheng Ma, Dan Wang, Wenxiao Dong, Sinan Wang, Tianyu Liu, Runxiang Xie, Li Liu, Bangmao Wang, Hailong Cao

**Affiliations:** ^1^Department of Gastroenterology and Hepatology, General Hospital, Tianjin Medical University, Tianjin, China; ^2^Department of Gastroenterology and Hepatology, Tangshan Gongren Hospital, Tangshan, Hebei Province, China; ^3^Department of Pathology, General Hospital, Tianjin Medical University, Tianjin, China

## Abstract

Inflammatory bowel disease (IBD), characterized by sustained inflammation, is a latent risk factor of colon tumorigenesis. Silibinin has been reported to be anti-inflammatory and antineoplastic, but its efficacy on colitis-associated cancer (CAC) has not been reported. Interlukin-6/signal transducer and activator of transcription 3 (IL-6/STAT3) is the key signaling pathway involved in CAC. We evaluated the chemopreventive effect of silibinin on a CAC mouse model and determined its impact on IL-6/STAT3 signaling. Intestinal tumor cells (IMCE and HCT-116 cell lines) were also treated by graded concentration of silibinin, and cellular viability was determined. Silibinin (750 mg/kg/day) was administered to an azoxymethane/dextran sulfate sodium (AOM/DSS) C57BL/6 mouse model for 10 weeks by gavage. Body weight, colon length, and the amount and diameter of colon tumors were documented, respectively. Specimens were subjected to H&E staining for colitis and tumor scoring, immunohistochemical staining and terminal deoxynucleotidyl transferase dUTP nick end labeling for proliferation assessment, and immunofluorescent staining for intestinal mucosa barrier assessment. Production of inflammatory cytokines was determined by real-time PCR. IL-6/STAT3 pathway activation was evaluated through immunohistochemical staining and western blot. In the current study, silibinin significantly inhibited the viability of intestinal tumor cells. The production of inflammatory cytokines and the phosphorylation of STAT3 were both inhibited in intestinal tumor cells. Meanwhile, silibinin decreased the amount and size of tumors in AOM/DSS mice. Colitis and tumor scores were decreased accompanying with inhibition of colonic tumor cell proliferation and promotion of cellular apoptosis. Additionally, silibinin could reduce the production of inflammatory cytokines and attenuate the impairment of colonic mucosal barrier. Furthermore, STAT3 phosphorylation was significantly suppressed by silibinin. In conclusion, silibinin could protect against colitis-associated tumorigenesis in mice via inhibiting IL-6/STAT3, which showed promising chemopreventive potential of CAC.

## 1. Introduction

Inflammatory bowel disease (IBD) is becoming a global issue with accelerating incidence in newly industrialized countries during the past three decades. Although the incidence is stabilizing in developed countries, it still remains a burden to the public hygiene [1]. Several studies have confirmed that IBD patients are at a higher risk of developing colitis-associated cancer (CAC) than the general population [2–6]. Risk of developing CAC in IBD patients is positively relevant to disease duration and the severity of inflammation such as pancolitis [5, 7, 8]. These evidences suggest that there may be innate correlations between colitis and CAC. Although the widespread introduction of 5-ASA, corticosteroids, thiopurine, and TNF-*α* blockers into clinical practice significantly decreased the risk of major surgeries for IBD patients [9–12], high-quality evidences supporting the chemopreventive efficacy of these agents for CAC are either controversial or absent [13–18]. It is still inconclusive whether these drugs can prevent the malignant transformation of colitis. So ideal agents which can prevent CAC still remain to be investigated.

IBD is characterized by sustained mucosal inflammation, which contributes to tumor initiation and progression because it enhances oxidative stress, promotes epithelium proliferation, and supports angiogenesis [19, 20]. The molecular mechanisms by which cancer was triggered and promoted may differ between CAC and sporadic CRC. Although CAC and sporadic CRC share common genetic changes, including silencing of tumor suppressor genes and aberrant expression of oncogenes as well as genetic instability, the classical “normal mucosa-adenoma-carcinoma” sequence in sporadic CRC progression is not confirmed in CAC, which originates from inflamed mucosa and progresses in an “inflammation-dysplasia-carcinoma” sequence [19–22]. The IL-6/STAT3 pathway has been proved to be a crucial tumor promoter in CAC [23–27]. IL-6 is predominantly produced by macrophages and monocytes during acute inflammation and by T cells during chronic inflammation. It binds to membrane-bound IL-6 receptor (mIL-6R) or soluble IL-6 receptor (sIL-6R) to form a complex with corresponding receptors. Then, the complex interacts with glycoprotein130 (gp130) and activates the subsequent downstream molecules [23]. STAT3 can be activated through activating with gp130. STAT3 is involved in the modulation of cellular proliferation and cell cycle. Continuous STAT3 activation can stimulate cell proliferation and prevent apoptosis and consequently trigger tumorigenesis [24]. So agents targeting IL-6/STAT3 signaling pathway may hopefully contribute to the prevention of CAC.

As a natural polyphenolic flavonoid extracted from milk thistle, silibinin exhibits potent antioxidative, anti-inflammatory, antiproliferative, immunomodulatory, and antiangiogenesis activities [28–32]. In the past two decades, researches have explored the efficacy of silibinin in various cancer cell lines, including skin, prostate, and lung cancers [30, 33–41], and have also demonstrated its anticancer effects in colon cancer cell lines such as HT-29, LoVo, SW480, and COLO205 [42–45]. A study conducted by Velmurugan et al. revealed that Fischer 344 rats fed with silibinin exhibited decreased aberrant formation of crypt foci induced by AOM [46]. Moreover, polyps in *Apc*^Min/+^ mice fed with silibinin were also reduced [47]. Nevertheless, to our knowledge, evidences testifying the antineoplastic property of silibinin in CAC are still limited. In this study, we demonstrated the chemopreventive effects and studied associated mechanisms of continuous silibinin intervention on an AOM/DSS mouse model and intestinal tumor cell lines.

## 2. Materials and Methods

### 2.1. Cell Culture

HCT-116 cells, purchased from the American Type Culture Collection (ATCC, Manassas, USA), were cultured in DMEM medium (Gibco, Invitrogen Corporation, NY, USA) supplemented with 10% heat-inactivated fetal bovine serum (FBS) (Gibco, Invitrogen Corporation, NY, USA), 100 U/ml benzylpenicillin, and 100 *μ*g/ml streptomycin with a pH of 7.4. The cells were maintained in a humidified atmosphere with 5% CO_2_ at 37°C. Immorto-Min colonic epithelial (IMCE) cell line, stemming from colonic epithelia of the hybrid between *Apc*^min/+^ mice and Simian vacuolating virus 40 (SV40) large T antigen transgenic mice, carried both the *Apc* gene and SV40 genome and was considered to be a premalignant cell line [48]. This cell line was kindly provided by Professor Fang Yan from Vanderbilt University. IMCE cells were cultured in RMPI 1640 medium (Gibco, Invitrogen Corporation, NY, USA) supplemented with 10% FBS, 0.05% interferon-*γ*, 100 U/ml benzylpenicillin, and 100 *μ*g/ml streptomycin under a circumstance of 33°C with 5% CO_2_. Silibinin (purity over than 99%) obtained from Tianjin Tasly Sants Pharmaceutical Co. Ltd. (Tianjin, China) was dissolved in DMSO to 0.1 M and stored at −20°C. When needed during experiments, the solution would be diluted with corresponding culture medium to gradient concentrations from 50 *μ*M to 800 *μ*M. The concentration of silibinin was confirmed by Raina et al. to efficiently inhibit the viability of CRC cells [49]. After being diluted, the concentration of DMSO was lower than 0.1% and had no impacts on cellular viability and differentiation. Both cell lines were transferred to 6-well plates to adhere and reach about 70% confluence and then starved for 15 h before being stimulated by silibinin and/or 10 *μ*g/ml lipopolysaccharide (LPS) (Sigma-Aldrich, St. Louis, MO, USA). Cellular lysates were collected for real-time PCR at 24 h and western blot assays at 3 h, 6 h, 12 h, and 24 h, respectively.

### 2.2. MTT Assay

The cells were seeded in a 96-well plate with a density of 5 × 10^3^/well for 24 h. Then, they were intervened with graded concentrations of silibinin from 50 to 800 *μ*M for 72 h. After the intervention, 10 *μ*l of 0.5 mg/ml MTT [3-(4,5-dimethylthiazol-2-yl)-2,5-diphenyltetrazolium bromide] (Sigma-Aldrich, St. Louis, MO, USA) solution was added for 4 h. Then, the MTT solution and medium were removed and 100 *μ*l DMSO was added to each well. An ELISA microplate reader was applied to determine cellular viability via measuring the absorbance at 570 nm.

### 2.3. Animals and Diets

Six-week-old female C57BL/6 mice, weighing 18–20 g, were purchased from Beijing Animal Study Center. The mice were housed under a specific pathogen-free environment at 22°C and 60% humidity, with a 12 h light and 12 h dark cycle for 7 days to acclimatize to the circumstances. They were fed with AIN-93M diet [50–52] and sterile water. CAC mouse model was induced by azoxymethane (AOM) and dextran sulfate sodium (DSS). Female C57BL/6 mice were randomly divided into 3 groups: control (*n* = 5), AOM/DSS (*n* = 15), and AOM/DSS/silibinin (*n* = 15). AOM (10 mg/kg) was injected intraperitoneally on day 0 (7 week old). On the same day, the mice were given 2% DSS in drinking water for 7 days, followed by 2 weeks of AIN-93M diet and water. There are a total of three cycles of the treatment (7 days DSS+ 14 day normal water) followed by a terminal week of normal water. From day 0, silibinin was administered by gavage (750 mg/kg body weight dissolved in 0.5% carboxymethyl cellulose (CMC)) every day until the end of the experiment. This dosage of silibinin was previously described by Rajamanickam et al. [47] and Ravichandran et al. [53]. This dosage has been proved to be of potent chemopreventive efficacy upon intestinal tumorigenesis, and our preliminary experiments certified its efficacy. The same volume of CMC was administered to the control group. Tumor development was evaluated on day 70. All the animals were fasted overnight before sacrifice. All the experimental procedures were conducted according to the guidelines of the Institutional Animal Care and Use Committee at Tianjin Medical University, Tianjin, China.

### 2.4. Tissue Collection and Measurement of Tumors

Mice were euthanized by excessive CO_2_. The whole colon was immediately excised, and the length and weight of the colon were measured and documented. Then, they were opened along the antimesenteric side and the contents were rinsed with sterile PBS solution. The amount of tumor in each colon was counted. The size of each tumor was measured using an Olympus SZX7 stereo dissecting microscope and classified as small (<2 mm), medium (2–4 mm), or large (>4 mm). Half of the colon tissue was snap frozen in liquid nitrogen. The other half was Swiss-rolled and fixed in 10% neutral-buffered formalin and later embedded in paraffin for the preparation of tissue slices. Tissue sections were later stained with hematoxylin and eosin (H&E) or subjected to immunohistochemical staining.

### 2.5. H&E and Immunohistochemical Staining

Formalin-fixed, paraffin-embedded colonic specimens were cut into 4 *μ*m slices for staining. Afterwards, tissue sections were deparaffinized in xylene and rehydrated in gradient ethanol. Then, slices were immersed in 3% hydrogen peroxide for 10 min to quench endogenous peroxidase activity. Antigens were restored in Antigen Unmasking Solution (Vector Laboratories Inc. Burlingame, CA, USA) for 15 min. Five percent goat serum dissolved in Tris-buffered saline was utilized to block nonspecific binding for 1 h at room temperature before H&E and immunohistochemical staining. Expression of Ki-67, IL-6, STAT3, p-STAT3, and F4/80 was detected, respectively, by incubating tissue sections with primary antibodies rabbit anti-Ki-67 (ab16667, Abcam, Cambridge, MA, USA), polyclonal rabbit anti-IL-6, monoclonal rabbit anti-p-STAT3 (Tyr705 phospho-STAT3), anti-STAT3 (9D8, Thermo Scientific, Beverly, MA, USA), anti-F4/80, anti-CD68, and rabbit anti-MUC2 (Santa Cruz Biotechnology Inc.) overnight at 4°C, respectively. Washed sections were incubated with corresponding horseradish peroxidase- (HRP-) labeled secondary antibodies at 37°C for 30 min followed by 3,3′-diaminobenzidine incubation for color development. All sections were observed by the same pathologist (DW) blind to our research using a light microscope. H&E-stained sections were examined and subjected to a histological injury score (HIS), a modified scoring system used by Kennedy et al. [54] (Table 1). Histological scores ranging from 0 to 15 were applied to each specimen according to the severity of inflammation. Corresponding to the different morphological presentation of tumors, these sections were scored as normal, 0; low-grade dysplasia, 1; high-grade dysplasia, 2; or invasive adenocarcinoma, 3. At least five fields of immunohistochemical-stained sections from each group were observed to calculate the number of positive cells, and the positive rate was calculated as the ratio of the amount of positive cells to the total amount of cells in each field. The positive rate of a certain group was represented as the average of all the five fields. To be specific, the positive rate of Ki-67 and p-STAT3 was determined by counting stained nuclei while cytoplasmic STAT3 staining was also added up to quantify its positive rate, and intercellular IL-6 staining was summed in each field to ascertain the positive rate. F4/80-stained cells in each crypt were counted, and the ratio of positive cells to the total cells in one certain crypt was calculated. At least 100 crypts per mouse were observed to calculate the average ratio, and the positive rate of a certain group was calculated as the average of each mouse in this group.

### 2.6. TUNEL Assay

Terminal deoxynucleotidyl transferase dUTP nick end labeling (TUNEL) assay was conducted to visualize apoptotic cells in colon tumors. Paraffin-embedded sections were deparaffinized and an in situ cell death detection kit (Roche Diagnostics, Basel, Switzerland) was applied to apoptotic nuclei staining according to the manufacturer's directions. To quantify cellular apoptosis, five fields randomly selected from every group were viewed.

### 2.7. Immunofluorescence Assay

The distribution of zonula occludens-1 (ZO-1) (Abcam, Cambridge, MA, USA) was visualized via immunofluorescence assay. Paraffin-embedded sections were deparaffinized, hydrated, and subjected to antigen retrieval. Five percent bovine serum albumin (BSA) (Solarbio, Beijing, China) was utilized to block nonspecific binding. Then, the sections were incubated with specific primary anti-ZO-1 antibody overnight at 4°C. Later, these sections were rinsed with PBS and incubated with Alexa Fluor 488 (FITC) secondary antibody in the dark for 60 minutes at room temperature. 4,6-diamidino-2-phenylindole (DAPI) (Sigma-Aldrich, St. Louis, MO, USA) was later applied to dye the nuclei. Fluorescence photographs were obtained under a fluorescence microscope DM5000 B (Leica, Wetzlar, Germany). FITC photos and DAPI photos were shot in the unified fields and merged by the Leica LAS AF Lite software version 2.3.

### 2.8. Periodic Acid Schiff (PAS) Staining

Deparaffinized colonic sections were incubated with 1% periodic acid solution (Sigma-Aldrich, St. Louis, MO, USA) for 10 min and later with Schiff reagent (Sigma-Aldrich, St. Louis, MO, USA) for 40 min. After that, these slices were counterstained with hematoxylin for 2–5 min. Each well was rinsed by PBS solution between every step.

### 2.9. Real-Time Polymerase Chain Reaction (PCR) Analysis

Total RNA was extracted from HCT-116 and IMCE cells and tumor-adjacent tissues utilizing the RNeasy mini kit (Qiagen, Carlsbad, CA, USA) and conversely transcribed using the TIANScript reverse transcription kit (TIANGEN Inc. Beijing, China), respectively. Real-time PCR was conducted to quantify the production of cytokines such as IL-6, IL-1*β*, TNF-*α*, and MUC2. Relevant oligonucleotide primer sequences were shown in Table 2. Glyceraldehyde-3-phosphate dehydrogenase (GAPDH), known as a housekeeping gene, was used as inner control to normalize the relative expression of targeted genes at mRNA level. Real-time PCR was performed using a StepOnePlus real-time PCR instrument (Applied Biosystems, Carlsbad, CA) following the manufacturer's directions. All cDNA products were assessed in triplicate. Expression of each transcript was quantified using the standard ∆∆CT method to calculate the fold changes normalized to corresponding internal controls. The procedure of PCR was constituted of 30 cycles followed by a period of 5 min at 72°C for final extension. Within each cycle, the time period and temperature were 94°C for 30s, 60°C for 30s, and 72°C for 90s, respectively. Relative expression of the genes was calculated using the 2^-ΔΔCT^ method.

### 2.10. Western Blot Analysis

Colon tumors were excised and stored at −80°C. Lysate of tumors and cellular lysate of IMCE and HCT-116 were prepared by sonication and RIPA buffer. Proteinase inhibitor cocktail (10 *μ*l/ml) (Sigma, St. Louis, MO, USA) and phosphatase inhibitor cocktail (10 *μ*l/ml) (Sigma, St. Louis, MO, USA) were added separately. After that, the lysate was homogenized and centrifuged (12,000 g, 4°C, 15 min). Then, the protein was separated by SDS-polyacrylamide gel electrophoresis before being transferred onto a PVDF membrane. Rabbit polyclonal antibodies (Abcam, Cambridge, MA, USA), anti-p-STAT3 and anti-STAT3, were adopted to conduct western blot and later blotted with secondary antibodies (anti-rabbit IgG peroxidase conjugates). *β*-Actin was selected as internal control to estimate the overall protein load in cellular lysate. Chemiluminescent signal of the PVDF membrane was detected by ECL (GE Healthcare, Bucks, UK) and visualized by forming image onto X-ray films. Comparison between the intensity of targeted bands and the intensity of internal control band was achieved via an image processor program (ImageJ).

### 2.11. Statistical Analysis

All continuous variables were described as mean ± SD. Statistical analyses of the multiplicity of colon tumors were performed using two-tailed Student's *t*-test using GraphPad Prism version 7.00. Positive staining rate and the ratio of the relative density of protein bands were compared via Student's *t*-test, respectively. Student's *t*-test was also adopted to compare differences of body weight between the AOM/DSS and AOM/DSS/silibinin group. *P* value less than 0.05 was deemed to be statistically significant.

## 3. Results

### 3.1. Silibinin Ameliorated Colitis and Tumor Load in AOM/DSS Mice

**Figure 1:**
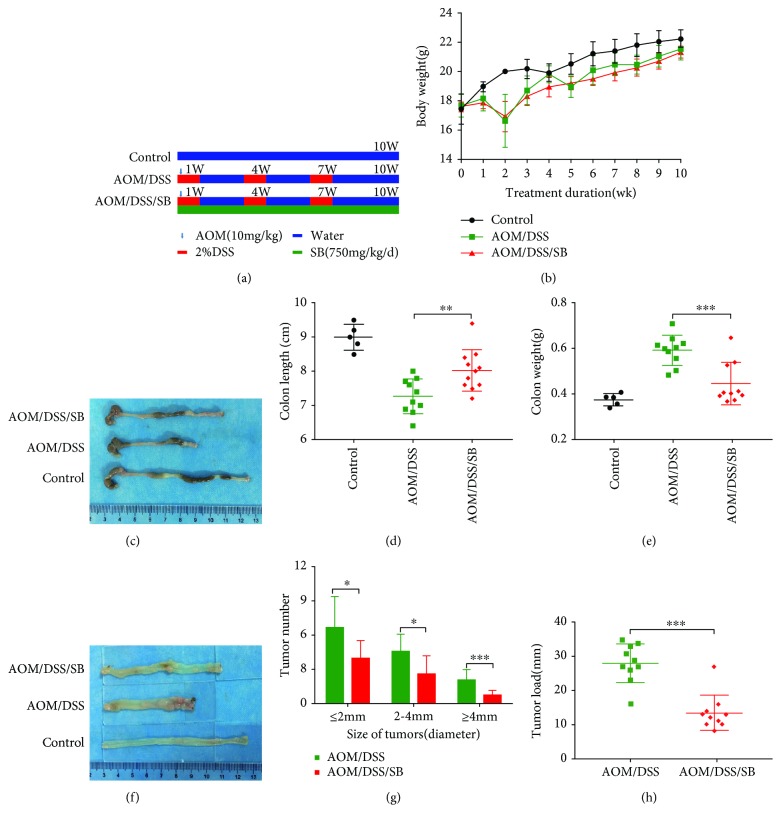
Silibinin treatment ameliorated colitis and tumor load in AOM/DSS mice. (a) The experimental course of AOM/DSS mouse model. (b) Body weight changes of all groups after treatment. (c) Macroscopic appearance of colon in the control, AOM/DSS, and AOM/DSS/SB groups. (d) Colon length after treatment at day 70. (e) Colon weight after AOM/DSS induction at day 70. (f) Colon was opened longitudinally after sacrifice and the amount of tumors were calculated. (g, h) Histogram showing the size distribution of tumors and average tumor load. SB: silibinin. *n* = 5~11 (^∗^*P* < 0.05, ^∗∗^*P* < 0.01, ^∗∗∗^*P* < 0.001, AOM/DSS vs. AOM/DSS/SB).

The CAC mouse model was induced by intraperitoneal injection of AOM followed by 3 cycles of DSS exposure (Figure 1(a)). Silibinin (750 mg/kg daily) did not affect the survival rate of mice, and 5 mice died (*n* = 15 − 5) (3 at week 2, 1 at week 4, and 1 at week 7) before the termination of the experiment in the AOM/DSS group, while 4 mice from the silibinin group died (*n* = 15 − 4) (2 at week 2 and 2 at week 3) during the experiments (*P* > 0.05). Mice receiving AOM/DSS lost some body weight at the end of each cycle, especially after the first cycle. Body weight was gradually restored within the period of normal water in the first two cycles, although the mice fell back to their initial body weight after the third DSS cycle. There was no significant difference in body weight loss between treatment with silibinin and without silibinin (*P* > 0.05) (Figure 1(b)).

It was observed that colon length challenged by AOM/DSS was evidently shorter in comparison with that in the AOM/DSS/silibinin group (*P* < 0.01) (Figures 1(c) and 1(d)). As expected, several colonic tumors were seen in all mice receiving AOM/DSS. Silibinin treatment significantly reduced the incidence and size of lesions (Figures 1(f) and 1(g)). There were significant differences in tumor size between the AOM/DSS and AOM/DSS/silibinin group (Figure 1(g)). Colon weight of AOM/DSS mice was heavier than that treated with silibinin at day 70 (*P* < 0.001) (Figure 1(e)). Correspondingly, mean tumor load, which was defined as the sum of diameters of all tumors in a certain mouse, was reduced in silibinin-treated mice (Figure 1(h)) (*P* < 0.001).

### 3.2. Silibinin Treatment Suppressed Colitis-Associated Colon Tumorigenesis

**Figure 2:**
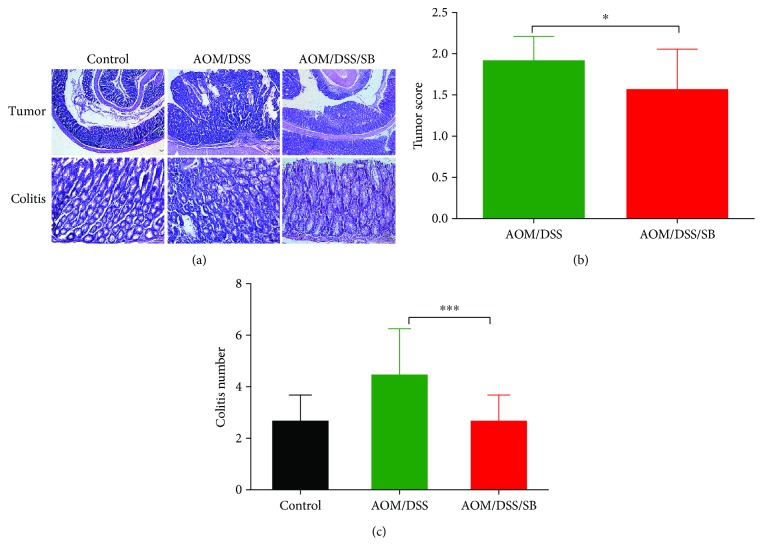
Silibinin treatment suppressed colitis-associated tumorigenesis. (a) Hematoxylin and eosin (H&E) staining of colon tumor and colitis in the control, AOM/DSS, and AOM/DSS/SB groups. Scale bars, 50 *μ*m. (b, c) Tumor score and colitis score of each group. SB: silibinin. *n* = 5~11 (^∗^*P* < 0.05, ^∗∗∗^*P* < 0.001, AOM/DSS vs. AOM/DSS/SB).

Hematoxylin and eosin (H&E) staining showed that silibinin inhibited the progression of CAC induced by AOM/DSS (Figure 2(a)). These differences demonstrated that silibinin showed apparent suppressive efficacy on AOM/DSS-induced colitis and colorectal tumorigenesis. To be exact, the proportion of high-grade dysplasia in the silibinin group is smaller than that in the AOM/DSS group (90% vs. 55%, *P* < 0.05), and there was a significant difference between the two groups in tumor score (*P* < 0.05) (Figure 2(b)). Additionally, colitis score of the silibinin-treated group was lower than that of the AOM/DSS group (*P* < 0.001) (Figure 2(c)).

### 3.3. Silibinin Decreased the Production of Inflammatory Cytokines in Intestinal Tumor Cells as well as the Colon of CAC Mice

**Figure 3:**
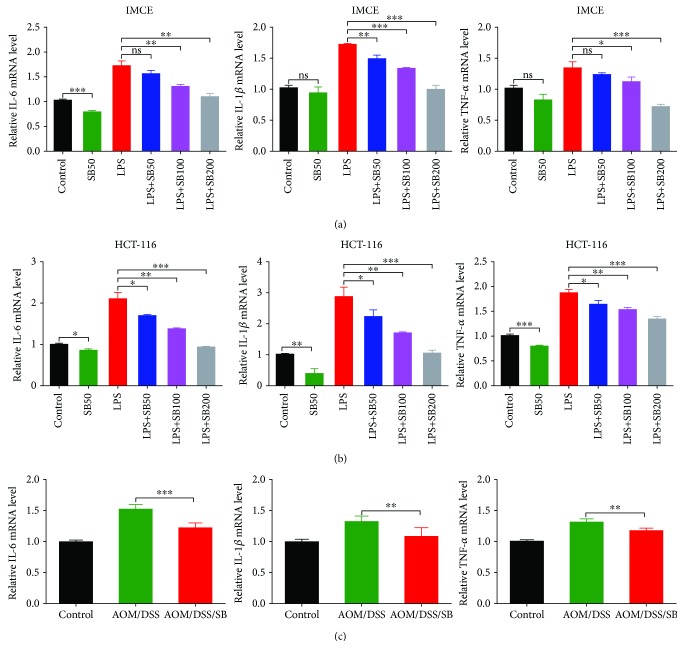
Silibinin decreased inflammatory cytokine production in intestinal tumor cell lines and AOM/DSS mice. (a, b) Real-time PCR was performed to detect the inflammatory cytokines in intestinal tumor cells. Relative expressions of IL-6, IL-1*β*, and TNF-*α* in IMCE (a) and HCT-116 cells (b) were presented. (c) Relative expression of IL-6, IL-1*β*, and TNF-*α* in colonic tumor-adjacent tissue in the control, AOM/DSS, and AOM/DSS/SB groups. Data were representative of three independent experiments and expressed as mean ± SD. SB: silibinin; ns: not significant. *n* = 5~11 (^∗^*P* < 0.05, ^∗∗^*P* < 0.01, ^∗∗∗^*P* < 0.001, AOM/DSS vs. AOM/DSS/SB).

IMCE and HCT-116 cells were incubated with gradient concentration of silibinin in the absence or presence of LPS for 24 h. The mRNA levels of proinflammatory cytokines including IL-6, IL-1*β*, and TNF-*α* in LPS-stimulated cells were significantly suppressed by multiple concentration of silibinin (Figures 3(a) and 3(b)). Together, the IL-6, IL-1*β*, and TNF-*α* productions were also significantly downregulated by silibinin in the AOM/DSS model (Figure 3(c)). These data suggested that the inhibition of inflammation by silibinin played a pivotal role in the prevention of colon tumorigenesis.

### 3.4. Silibinin Inhibited Cell Proliferation and Apoptosis in Intestinal Tumor Cells and Tumors of CAC Mice

**Figure 4:**
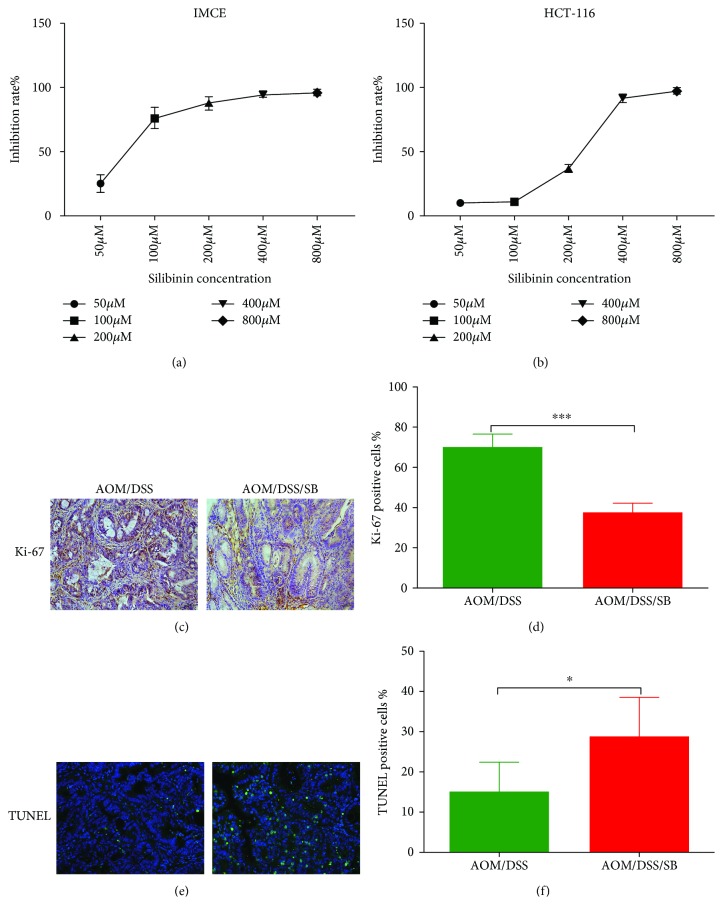
Silibinin supplementation inhibited proliferation and promoted apoptosis in intestinal tumor cells. (a, b) IMCE and HCT-116 cells were treated with silibinin at indicated concentrations for 72 h, respectively. Cell viability was then determined by MTT assay. Data are representative of three independent experiments and expressed as mean ± SD. (c, d) Colon sections from the AOM/DSS and AOM/DSS/SB groups were stained with Ki-67 (brown staining). (e, f) Colon sections from the two groups were stained with TUNEL. Green staining represented apoptotic cells. Scale bars, 50 *μ*m. Positive rate was determined by counting positively stained nuclei in tumor cells at 5 randomly selected fields from each group. SB: silibinin. *n* = 5~11 (^∗^*P* < 0.05, ^∗∗∗^*P* < 0.001, AOM/DSS vs. AOM/DSS/SB).

To explore the antitumor activities of silibinin, MTT assay was employed to assess their effects on cell viability in the IMCE cell line and HCT-116 colorectal cancer cells. Silibinin concentration-dependently decreased the viability of IMCE and HCT-116 cells after 72 h exposure. The individual IC50 of silibinin was approximately 250 *μ*M in HCT-116 cells and 75 *μ*M in IMCE cells (Figures 4(a) and 4(b)). These results suggested that silibinin had potent antiproliferative effects in premalignant and cancer cells, and interestingly the effects were more pronounced in premalignant cells than in cancer cells. Since 100 *μ*M silibinin was enough to exert significant inhibitory efficacy towards the viability of both HCT-116 and IMCE cell lines according to our results, this dosage was selected to stimulate cells in western blot analysis.

Cell proliferation and apoptosis were visualized and quantified to evaluate colon tumor development in mice. There was a dramatic decrease in the amount of proliferative cells within tumors of silibinin-treated mice, compared with those of AOM/DSS mice (*P* < 0.001) (Figures 4(c) and 4(d)). The silibinin group showed more apoptotic cells than the AOM/DSS group (*P* < 0.05) (Figures 4(e) and 4(f)).

### 3.5. Silibinin Restored the Impaired Colon Mucosal Barrier and Alleviated the Infiltration of Macrophages

**Figure 5:**
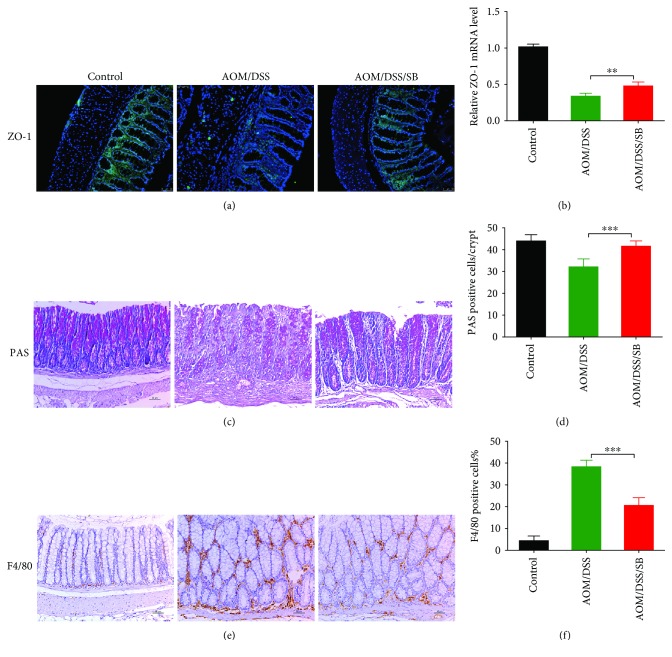
Silibinin supplementation restored colonic barrier function and ameliorated the infiltration of macrophages. (a) Paraffin-embedded specimens of colonic tumor-adjacent tissue were subjected to immunofluorescent staining for ZO-1 distribution using an anti-ZO-1 antibody and a FITC-labeled secondary antibody (green staining). Nuclei were stained with DAPI (blue staining). (b) Real-time PCR analysis of ZO-1 expression in colonic epithelium was also shown. (c, d) PAS staining for goblet cells in tumor-adjacent tissue was shown, and diagrams presented average positive cells per crypt of each group. (e, f) Expression of F4/80 in tumor-adjacent tissue was assessed using immunohistochemical staining. The positive rate was determined by counting the absolute number of positive staining in at least 100 colonic crypts of each mouse. Scale bars, 50 *μ*m. SB: silibinin. *n* = 5~11 (^∗∗^*P* < 0.05, ^∗∗∗^*P* < 0.01, AOM/DSS vs. AOM/DSS/SB).

Immunofluorescent staining was conducted to estimate the distribution of ZO-1, essential constituents of tight junction protein. The AOM/DSS group was accompanied with an impaired tight junction, while silibinin supplementation partly restored the impaired epithelial tight junction (Figures 5(a) and 5(b)). Goblet cells visualized by PAS staining severely decreased after AOM/DSS challenge whereas silibinin supplement significantly protected them from AOM/DSS destruction (Figures 5(c) and 5(d)). Our results manifested that silibinin might be efficacious in promoting the differentiation of goblet cells and enhancing the integrity of intestinal barrier.

As essential constituents of intestinal immune barrier, macrophages play a crucial role in the modulation of tumor microenvironment [55]. F4/80 is a specific immunomarker of macrophages [56, 57]. In the current study, a dramatic enhancement in F4/80 expression in colonic tumor-adjacent tissue of the AOM/DSS group was observed. In the silibinin group, however, F4/80 expression was significantly lower than that in the AOM/DSS group (*P* < 0.001) (Figures 5(e) and 5(f)), which further supported that silibinin could moderate the infiltration of intestinal macrophages in mice.

### 3.6. Silibinin Treatment Downregulated IL-6/STAT3 Pathway in Intestinal Tumor Cells and Colon Tissues of AOM/DSS Mice

**Figure 6:**
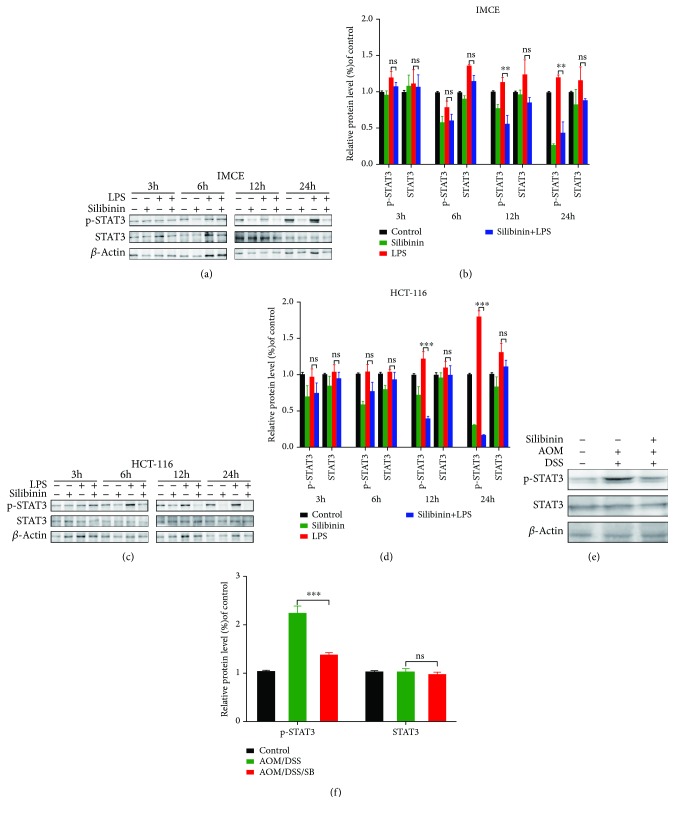
Silibinin inhibited STAT3 phosphorylation in intestinal tumor cells. (a) Western blot showed the expression of p-STAT3 and STAT3 in IMCE cells after incubation with silibinin and/or LPS for 3 h, 6 h, 12 h, and 24 h, respectively. (b) ImageJ was applied to densitometric analyses to determine the expression of p-STAT3 and STAT3 in IMCE cells, and the data were normalized as relative ratio to *β*-actin. (c) The expression of p-STAT3 and STAT3 in HCT-116 cells after incubation with silibinin and/or LPS for 3 h, 6 h, 12 h, and 24 h, respectively. (d) Densitometric analyses to determine the expression of p-STAT3 and STAT3 in HCT-116 cells. (e) Western blot was also utilized to show the expression of p-STAT3 and STAT3 in the control, AOM/DSS, and AOM/DSS/SB groups. (f) Densitometric analyses were also involved in quantifying the expression of p-STAT3 and STAT3 in the control, AOM/DSS, and AOM/DSS/SB groups. SB: silibinin; ns: not significant. *n* = 5~11 (^∗∗^*P* < 0.01, ^∗∗∗^*P* < 0.001, AOM/DSS vs. AOM/DSS/SB).

**Figure 7:**
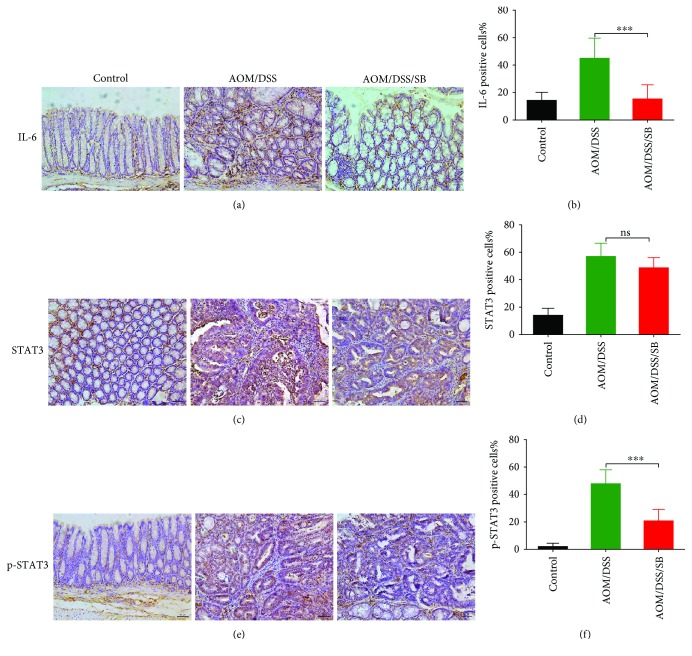
Silibinin downregulated IL-6/STAT3 pathway in AOM/DSS mice. (a, b) The expression of IL-6 in colonic tissues from the control, AOM/DSS, and AOM/DSS/SB mice was visualized by immunohistochemical staining. The percentage of IL-6 positive staining in five randomly selected fields of each group was shown. (c, f) STAT3 (c) and p-STAT3 (e) in colonic tissues from the three groups were detected. Positive rate of STAT3 (d) and p-STAT3 (f) in five randomly selected fields of each group was also presented separately. Scale bars, 50 *μ*m. SB: silibinin; ns: not significant. *n* = 5~11 (^∗∗∗^*P* < 0.001, AOM/DSS vs. AOM/DSS/SB).

Silibinin could inhibit LPS-induced cellular STAT3 phosphorylation *in vitro*. The suppressive effect was not significant until 12 h after silibinin supplement. Cellular STAT3 phosphorylation was even completely blocked after 24 h silibinin coincubation while the total expression of STAT3 was not significantly changed (Figures 6(a)–6(d)). *In vivo*, STAT3 phosphorylation was blocked in tumors of the silibinin group compared with that of the AOM/DSS group (*P* < 0.001) (Figures 6(e) and 6(f)). The inhibitory effect of silibinin was further validated by immunohistochemical staining. Immunohistochemical analysis presented strong expression of IL-6 and p-STAT3 in intestinal epithelia of the AOM/DSS group and much lower expression in normal colonic mucosa (Figures 7(a), 7(b), 7(e), and 7(f)). In contrast, CAC mice treated with silibinin exhibited a significant decrease in the production of IL-6 and p-STAT3 in colon tissue compared with the AOM/DSS group while total production of STAT3 was not affected (Figures 7(c) and 7(d)). All these results indicated that the antineoplastic property of silibinin might be at least partially attributed to suppression upon IL-6/STAT3 signaling pathway.

## 4. Discussion

Epidemiological studies have already pointed out that up to 18.4% IBD patients may have CRC three decades after their colitis was diagnosed [58]. Given the accumulatively ascending incidence of IBD in newly industrialized countries and the maintenance of high incidence in developed countries [1], it is essential to prevent tumorigenesis among IBD patients. 5-ASA, corticosteroids, thiopurine, and TNF-*α* blockers, which are extensively used to treat IBD in clinical practice, were verified by large cohort studies to significantly reduce or delay the requirement for major surgeries [9–12]. Regretfully, whether these drugs can prevent the trigger and progression of CAC still remains inconclusive [13–18]. Chemopreventive efficacy of herbal agent has been attracting more attention during the past decade [59, 60], which enlightened us to investigate the chemopreventive potential of herbs. In our study, we demonstrated that silibinin, a traditional herbal medicine, could suppress the viability of intestinal tumor cells and inhibit intestinal inflammation and tumorigenesis induced by AOM/DSS. In addition, the intestinal barrier function was restored with silibinin supplement. We also investigated possible mechanisms related to IL-6/STAT3 signaling pathway downregulated by silibinin both *in vivo* and *in vitro*. Thus, our findings suggested that silibinin might be efficacious against CAC.

Lately the antineoplastic activity of silibinin in CRC rodent models has been reported. Silibinin decreased proliferating cell nuclear antigen (PCNA) and cyclin D1 expression and increased cyclin-dependent kinase-interacting protein 1 (Cip1)/p21 expression in A/J mice administered with AOM, which indicates its antiproliferative and proapoptotic effect [53]. Kauntz et al. also testified the efficacy of silibinin on a Wistar rat model. Colon from silibinin-administered rats showed a 2-fold decrease in the amount of hyperproliferative crypts and aberrant crypt foci compared with that from their AOM-injected counterparts. Furthermore, silibinin intensified the activity of caspase-3 and downregulated the production of antiapoptotic protein B-cell lymphoma-2 (Bcl-2) [61]. Our study demonstrated that silibinin ameliorated colitis and relieved the symptoms in AOM/DSS mice. Furthermore, administration of silibinin not only repressed the proliferation of tumors but also enhanced the apoptosis, resulting in significant difference in tumor load. To our knowledge, this is the first study focusing on the efficacy of silibinin using both intestinal tumor cell lines and a CAC mouse model. Consistent with previous studies on CRC animal models and cell lines, our results confirmed that silibinin could also inhibit tumorigenesis on CAC animal models and premalignant cell lines despite the differences in the mechanisms of CRC and CAC. Notably, the MTT assay indicated that the antineoplastic effects were more pronounced in premalignant cells than in cancer cells. This phenomenon suggests that silibinin can be applied not only in the treatment of neoplasm but also in the prevention.

It has been revealed that defective intestinal barrier is correlated to CRC development. Interestingly, 65% of *Muc2*^−/−^ mice spontaneously formed intestinal tumors at the age of 12 months. Intestinal specimens of *Muc2*^−/−^ mice exhibited absent staining for goblet cells and higher cellular proliferation with decreased apoptosis compared with those of wild-type mice [62]. PAS staining and immunohistochemical staining of human CRC tissue also showed the absence of goblet cells and MUC2, while both the amount of goblet cells and the secretion of MUC2 were normal in the tumor-adjacent tissue [63]. Sangeetha and Nalini manifested that silibinin could attenuate CRC induced by 1,2-dimethyl hydrazine in experimental rats, and mucin secretion was depleted by 1,2-dimethyl hydrazine administration while silibinin ameliorated the depletion [64]. Similarly, our data indicated that AOM/DSS severely damaged goblet cells, while silibinin supplement significantly restored goblet cells and ZO-1 protein. This implied that CAC is also accompanied by the destruction to intestinal barriers, and the restoration of intestinal barriers with silibinin supplement may partly contribute to its antineoplastic efficacy. Whether silibinin directly protected goblet cells from destruction or silibinin upregulated the differentiation from stem cells to goblet cells remains to be further investigated.

Studies have established the crucial role of IL-6/STAT3 axis in the progression of CAC [23, 24]. IL-6 production is elevated in IBD patients in both serum and intestinal mucosa specimens. Excessive activation of STAT3 mediated by increasing IL-6 enhances the expression of antiapoptotic factors such as Bcl-2, which contributes to the sustainability of chronic colitis [26]. Mice with IL-6 ablation exhibited reduced number, size, and multiplicity of tumors compared with wild-type counterparts after AOM/DSS exposure. Moreover, *Il6*^−/−^ mice exhibited elevated apoptosis and decreased proliferation in tumor specimens with lower cyclin D expression and Ki-67 positive rate [27]. STAT3 was also reported to be excessively phosphorylated in epithelial and lamina propria cells of IBD patients and in DSS-induced colitis mouse models [65, 66]. Mice with a specific deletion of STAT3 in enterocytes (*Stat3*^△IEC^) developed similar phenotypes to that of *Il6*^−/−^ mice, which supported the contribution of STAT3 in CAC tumorigenesis. *Stat3*^△IEC^ mice were almost completely inhibited from the formation of AOM-induced tumor while the mouse model with STAT3 hyperactivation (*gp130*^Y757F^) had even more severe tumor load after AOM/DSS challenge [25]. In the current study, we proved that silibinin downregulated the production of IL-6 and inhibited the phosphorylation of STAT3 both *in vivo* and *in vitro*, which partly explained its inhibitory effect against CAC formation. We have not determined whether silibinin downregulates IL-6/STAT3 axis directly or through other mediators in the current study. Further researches are still needed to explain how silibinin modulates IL-6/STAT3 signaling pathway.

Taken together, our study demonstrated that silibinin administration ameliorated colitis and inhibited colitis-associated tumorigenesis via inhibition of IL-6/STAT3 signaling pathway. Our data might offer evidence for chemoprevention of CAC, which suggested that silibinin could be a promising option for CAC prevention and treatment.

## Figures and Tables

**Table 1:** Histological injury score.

Enterocyte loss	Normal	0
	Loss of single cell	1
	Loss of groups of cells	2
	Frank ulceration	3
Crypt inflammation	Normal	0
	Single inflammatory cell	1
	Cryptitis	2
	Crypt abscess	3
Lamina propria mononuclear cells	Normal	0
	Slight increase	1
	Moderate increase	2
	Marked increase	3
Neutrophils	Normal	0
	Slight increase	1
	Moderate increase	2
	Marked increase	3
Epithelial hyperplasia	Normal	0
	Mild	1
	Moderate	2
	Pseudopolyp	3

**Table 2:** Gene sequences of primers in the present study.

Primers	Sequences
Human	
GAPDH	Forward: 5′-AGGTCGGTGTGAACGGATTTG-3′
	Reverse: 5′-TGTAGACCATGTAGTTGAGGTCA-3′
IL-6	Forward: 5′-TAGTCCTTCCTACCCCAATTTCC-3′
	Reverse: 5′-TTGGTCCTTAGCCACTCCTTC-3′
IL-1*β*	Forward: 5′-GCAACTGTTCCTGAACTCAACT-3′
	Reverse: 5′-ATCTTTTGGGGTCCGTCAACT-3′
TNF-*α*	Forward: 5′-TTCTGCCTGCTGCACCTTGGA-3′
	Reverse: 5′-TTGATGGCAGAGAGGAGGTTG-3′
Mouse	
GAPDH	Forward: 5′-GAGTCAACGGATTTGGTCGT-3′
	Reverse: 5′-TTGATTTTGGAGGGATCTCG-3′
IL-6	Forward: 5′-AGCCAGAGTCCTTCAGAGAG-3′
	Reverse: 5′-ACTCCTTCTGTGACTCCAGC-3′
IL-1*β*	Forward: 5′-GTAATGAAAGACGGCACACCC-3′
	Reverse: 5′-GTGCTGATGTACCAGTTGGG-3′
TNF-*α*	Forward: 5′-TCGAGTGACAAGCCTGTAGC-3′
	Reverse: 5′-GGAGGTTGACTTTCTCCTGG-3′
ZO-1	Forward: 5′-GGGCCATCTCAACTCCTGTA-3′
	Reverse: 5′-AGAAGGGCTGACGGGTAAAT-3′

## Data Availability

The data used to support the findings of this study are available from the corresponding author upon request.
